# Supplementary material for: A Study on the Antibacterial, Antispasmodic, Antipyretic, and Anti-Inflammatory Activity of ZnO Nanoparticles Using Leaf Extract from Jasminum sambac (L. Aiton)
Source: Molecules. 2024 Mar 25;29(7):1464. doi: 10.3390/molecules29071464 (PMC11012760; doi:10.3390/molecules29071464)
Supplement: Supplementary file 1 [file molecules-29-01464-s001.zip › molecules-2906351-supplementary.pdf]

Supplementary material for:

Studies on the Antibacterial, Antispasmodic, Antipyretic, and Anti-inflammatory Activity of ZnO Nanoparticles Using Leaf Extract from *Jasminum sambac* (L. Aiton)

Primary metabolites of plants with an aromatic ring and at minimum one hydroxyl group are referred to as natural phenolic compounds. More than 8,000 plant-derived phenolic compounds have been discovered to date. A number pharmacological properties, like antioxidant, antibacterial, antiviral, anti-allergenic, cardioprotective, neuroprotective, and anticancer properties, are known to be present in these phytochemicals. In this method, it is suggested that the phenolic content of plant extracts acts as an essential biological characteristic indicator. The results of a qualitative phytochemical analysis of a number *Jasminum sambac* leaf extract is shown in Table S1.

Table S1. Qualitative phytochemical analysis of different extracts of *Jasminum sambac* (leaf extracts).

**S. No**	**Phytoconstituents**	**n-Hexane extract**	**Chloroform extract**	**Ethyl acetate extract**	**Methanol extract**	**Water extract**
**1.**	Glycosides	+	+	+	+	+
**2.**	Alkaloids	+	+	+	+	+
**3.**	Phytosteroids	-	-	+	-	-
**4.**	Flavonoids	+	+	+	+	+
**5.**	Saponins	+	+	+	+	+
**6.**	Tannins and	-	-	-	-	-

	phenolic compounds					
**7.**	Fixed oils and fats	-	-	-	-	-

(+) = indicates presence; (-) = indicates absence

Characterization:

The novelty of the current study is the investigation of the anti-bacterial, anti-spasmodic, anti-pyretic anti-inflammatory activity of synthesized ZnO NPs utilizing *Jasminum sambac* leaf extracts. The chemical component of *Jasminum sambac* leaf extract can be described by the analysis like FTIR, UV-visible spectroscopy, and SEM analysis.

FTIR analysis:

FTIR spectrum was observed at 3424, 2925, 1732 and 1453 cm^{-1}. The infrared spectra showed signals for two major functional groups identified as –OH (3400 cm^{-1}), –CH (2925 cm^{-1}), C=O (1732 cm^{-1}) and C=C (1453 cm^{-1}) corresponding to the chemical structure of saponins.

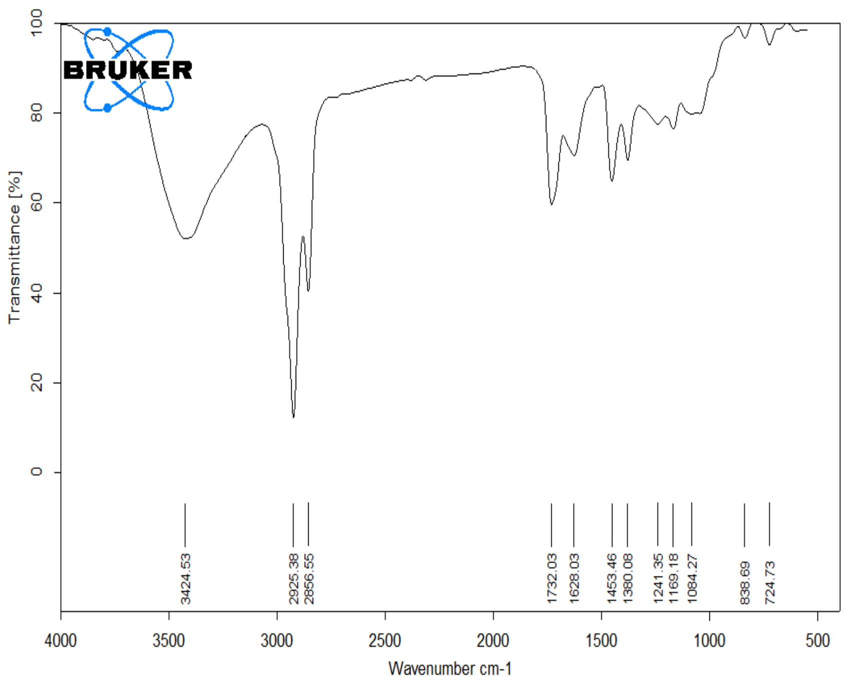

Figure S1. FTIR spectrum of *Jasminum sambac* leaf extract.

UV-visible spectroscopy analysis:

The UV-visible absorption spectra of the leaf extract of *Jasminum sambac* is shown in Figure S2, but the leaf extract of *Jasminum sambac* did not display any peaks. The leaf extract has a bump that measures between 200 to 300 nm; this could be due the extract has polyphenolic compounds.

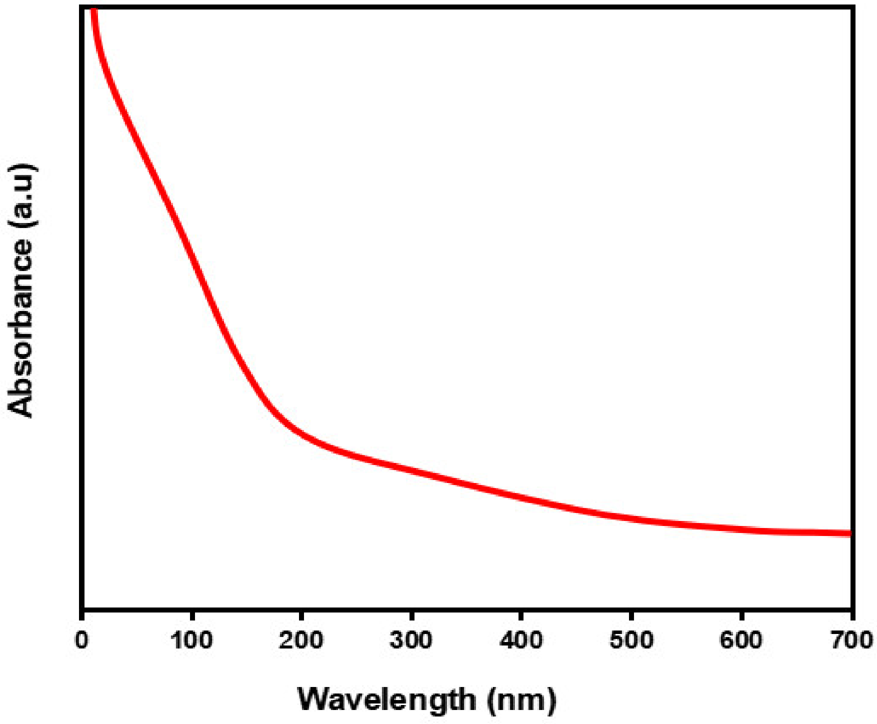

Figure S2. UV-visible spectra of *Jasminum sambac* leaf extract.

SEM analysis:

SEM analysis was performed on the morphology of the leaf extract of *Jasminum sambac*. SEM images of leaf extract were shown in the final results. The significant effects of the extraction on the extract solution were found.

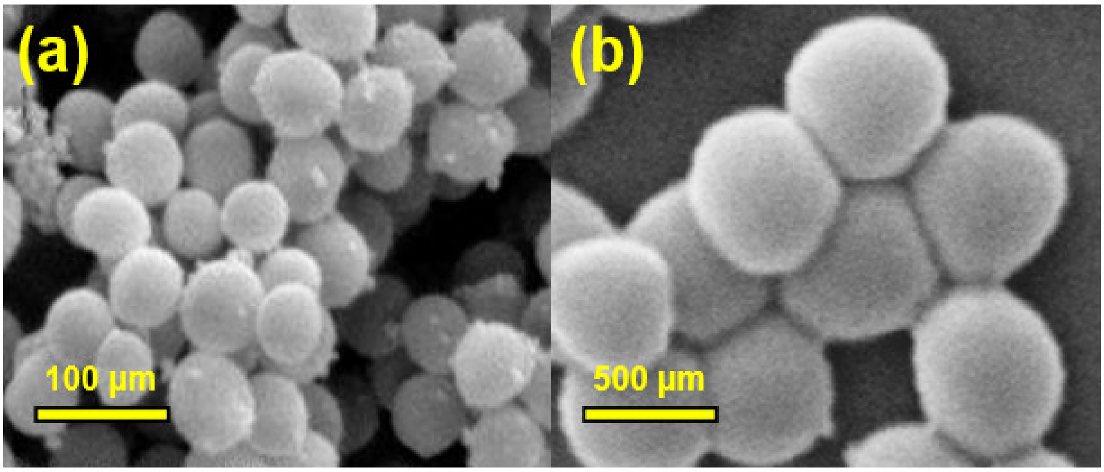

Spherical particles of the leaf extract of *Jasminum sambac* was observed.

Figure S3. SEM images of *Jasminum sambac* leaf extract (a, and b).
